# THE LONGEVITY CONSORTIUM VISION

**DOI:** 10.1093/geroni/igz038.758

**Published:** 2019-11-08

**Authors:** Daniel S Evans, Daniel S Evans, Steven R Cummings, Nicholas Schork

**Affiliations:** 1 California Pacific Medical Center Research Institute, San Francisco, California, United States; 2 California Pacific Medical Center Research Institute, UCSF, San Francisco, California, United States; 3 San Francisco Coordinating Center, CPMCRI, UCSF, San Francisco, California, United States; 4 TGen, Phoenix, Arizona, United States

## Abstract

Molecular factors and pathways promoting human longevity and healthy aging can potentially delay or prevent multiple chronic diseases and conditions, but identifying such factors that can be pharmacologically targeted requires an integrated multidisciplinary approach. We describe the design of the five research projects and three cores of the Longevity Consortium (LC) and how their cooperative research is designed to discover molecular factors and pathways that can predict healthy human aging and longevity, associate with extreme human lifespan, show relevance to chronic age-related conditions, respond to interventions to slow aging in mice, and show evidence for association with lifespan across species. A systems biology approach is undertaken to identify common molecular features across human traits and organisms, and a chemoinformatics approach to link molecular targets to candidate healthy aging interventions. The LC results are made publicly available and we provide funding opportunities to the scientific community to support pilot projects.

